# Synthesis and Characterization of Halloysite/Carbon Nanocomposites for Enhanced NSAIDs Adsorption from Water

**DOI:** 10.3390/ma12223754

**Published:** 2019-11-14

**Authors:** Beata Szczepanik, Nina Rędzia, Laura Frydel, Piotr Słomkiewicz, Anna Kołbus, Katarzyna Styszko, Tadeusz Dziok, Bogdan Samojeden

**Affiliations:** 1 Institute of Chemistry, Jan Kochanowski University, 25-406 Kielce, Poland; dziewit.n@gmail.com (N.R.); laura.frydel@gmail.com (L.F.); piotres@ujk.edu.pl (P.S.); anna.kolbus@ujk.edu.pl (A.K.); 2 The Structural Research Laboratory, Jan Kochanowski University, Swietokrzyska 15G, 25-426 Kielce, Poland; 3 Faculty of Energy and Fuels, AGH University of Science and Technology, Al. Mickiewicza 30, 30-059 Kraków, Poland; styszko@agh.edu.pl (K.S.); tadeusz.dziok@agh.edu.pl (T.D.); bogdan.samojeden@agh.edu.pl (B.S.)

**Keywords:** halloysite/carbon nanocomposites, non-steroidal anti-inflammatory drugs, adsorption

## Abstract

The adsorption of ketoprofen, naproxen, and diclofenac (non-steroidal anti-inflammatory drugs, NSAIDs) on halloysite/carbon nanocomposites and non-modified halloysite were investigated in this work. Halloysite/carbon nanocomposites were obtained through liquid phase impregnation and carbonization using halloysite as the template and saccharose as the carbon precursor. Scanning electron microscopy (SEM), X-ray diffraction (XRD), Fourier-transform infrared spectrometry (FT-IR), and low-temperature nitrogen adsorption method were employed to study the morphological and structural changes of the halloysite/carbon nanocomposites. The effects of contact time, initial concentration of adsorbates, pH of solution, and mass of adsorbent on the adsorption were studied. Adsorption mechanism was found to fit pseudo-second-order and intra-particle diffusion models. The obtained experimental adsorption data were well represented by the Langmuir multi-center adsorption model. Adsorption ability of halloysite/carbon nanocomposites was much higher for all the studied NSAIDs in comparison to non-modified halloysite. Optimized chemical structures of ketoprofen, naproxen, and diclofenac obtained by Density Functional Theory (DFT) calculation showed that charge distributions of these adsorbate molecules and their ions can be helpful to explain the details of adsorption mechanism of NSAIDs on halloysite/carbon nanocomposites.

## 1. Introduction

In recent decades, a variety of new chemical compounds have been identified in wastewater. These trace compounds, mostly organic (personal care products, endocrine disrupting compounds, pharmaceuticals, and their transformation products) are known as the “emerging contaminants”. Pharmaceuticals and personal care products (PPCPs) have been detected in surface water and wastewater in the nanogram/liter to micrograms/liter concentration range worldwide [1]. Active pharmaceuticals and their biotransformation products have a tendency for bioaccumulation [2] and can cause significant consequences to ecosystems [3,4]. Hence, it is important to develop efficient and economic methods for removing these compounds from wastewater. Several techniques have been studied, including oxidation, adsorption, electro degradation, bio-degradation, and photocatalytic degradation [5]. Adsorption is one of the most promising methods for removing organic micropollutants [6]. Adsorbents, including zeolites [7,8], activated carbon [9,10], biochar [11], activated hydrochars [12], carbon nanotubes [13], and composites with activated carbon [14], were used for PPCP removal from aqueous systems. Adsorbents used for ketoprofen, naproxen, and diclofenac removal from aqueous solutions are collected in Table 1. Carbon materials are often used for wastewater treatment because of their high specific surface area, abundant surface groups, and high stability [15]. However, although these carbon materials show good adsorption ability, the main drawback of using them results from a costly and complicated synthesis process. Compared with traditional carbon adsorbents, economical and effective composites with high adsorption ability can be received as a result of a combination of clay minerals and carbon. Naturally-occurring clay minerals can also act as adsorbents [16,17,18], so they can be used to enhance adsorption properties of carbon/mineral composites [19]. Biomass, such as glucose, cellulose, and fructose, can be converted to carbon materials through hydrothermal carbonization [20,21]. The montmorillonite–carbon composites used as adsorbents to remove 2,4,6-trichlorophenol and methylene blue from aqueous solutions were obtained with commercial table sugar as carbon source in the presence or absence of H_2_SO_4_ [22]. Anadao et al. used waste bleaching sodium montmorillonite clay to prepare carbon–clay nanocomposites. Adsorption capacities of the obtained composites were higher for methylene blue and gasoline adsorption than for montmorillonite as a result of higher carbon content in composite [19]. The attapulgite clay–carbon nanocomposite was synthesized by a one-pot hydrothermal carbonization process under mild conditions using attapulgite clay and glucose, as carbon source, obtained from biomass. This nanocomposite was used as adsorbent, which exhibits high adsorption ability for Cr(VI) and Pb(II) ions [23]. Wu et al. synthetized palygorskite/carbon composite by hydrothermal carbonization of glucose onto palygorskite using ammonium ferrous iron (Fe^2+^) sulphate hexahydrate as catalyst. This composite demonstrated high adsorption capacity for phenol in comparison with unmodified palygorskite [24]. Palygorskite/carbon composites were also prepared through hydrothermal carbonization using palygorskite clay and cellulose as carbon source. These composites were used for removing methylene blue and phenol from aqueous solutions [25,26].

Halloysite (Hal) is a 1:1 layered clay with one silica tetrahedron sheet and one alumina octahedron sheet clay. Naturally-occurring, low cost, tubular nanostructure (1D nanomaterial), good mechanical properties, excellent chemical stability, high specific surface area and porosity, and large adsorption capacity make it attractive for technology [27,28]. Wu et al. obtained halloysite–carbon nanocomposites via a hydrothermal route using halloysite as the template and cellulose as the carbon precursor. These nanocomposites exhibited a significantly higher phenol removal rate in comparison to common activated carbon and unmodified halloysite [29]. Halloysite nanotubes–Fe_3_O_4_–carbon nanocomposites (HNT–Fe_3_O_4_–C) were synthetized by Jiang et al. and applied as adsorbent for methylene blue removal from water [30]. Halloysite nanotubes–carbon nanocomposites were hydrothermally synthesized with sucrose as carbon source and used as a medium for physisorption-based hydrogen storage [31]. The halloysite–carbon nanocomposite from sucrose-coated was used as a carrier by Zhang et al. to produce ZnO- and TiO_2_-coated photocatalyst for photodegradation of methylene blue dye. The presence of carbon increased the adsorption of dyes and facilitated charge transportation [32]. Zhang et al. showed that HNTs/C nanocomposites can be applied as biocompatible nanomaterial for drug delivery carriers. They synthesized these nanocomposites by one-step hydrothermal carbonization of glucose on the surface of HNTs [33]. 

The novelty of our study is the first use of halloysite/carbon nanocomposites as adsorbents for the removal of pharmaceuticals such as NSAIDs from aqueous solutions.

In the present study, saccharose was used as a carbon precursor to synthesize halloysite/carbon nanocomposites through liquid phase impregnation and carbonization. The composition and structural characteristics of obtained composites were studied. Halloysite/carbon materials and non-modified halloysite were used as adsorbents of the chosen NSAIDs: Ketoprofen, naproxen, and diclofenac. Based on the knowledge available to us, we applied these composites to remove NSAIDs from an aqueous solution for the first time. 

## 2. Materials and Methods

### 2.1. Materials and Reagents

Modified halloysite was obtained from strip mine “Dunino”, Intermark Company, Legnica, Poland. Saccharose is a widely available commercial product, methanol (98%) was purchased from Chempur, Piekary Slaskie, Poland. Ketoprofen, diclofenac sodium, naproxen(S)-(+)-2-(6-Methoxy-2-naphthyl) propionic acid, 99% was acquired from Alfa Aesar, Germany, deionized water was used through all experiments. The selected physicochemical properties of the adsorbates are presented in Table 2.

### 2.2. Preparation of Halloysite/Carbon Composites

Modification of halloysite was carried out in the following manner: Raw halloysite (RH) was dried, powdered, and washed with deionized water. Next, the magnetic fraction containing Fe_2_O_3_ was separated using a 2-T magnetic separator. The obtained non-magnetic fraction of a halloysite mineral was cleaned with distilled water, dried, and followed by reductive bleaching with sodium hydrosulfite. Chemical composition of raw (RH) and modified halloysite (H) was published in ref. [35]. Particle fractions of 0.2–0.32 mm were used for the preparation of halloysite/carbon composites. Halloysite/carbon composites were prepared through liquid phase impregnation and carbonization. In detail, 20.0 g of H was added into 100 cm^3^ of saccharose aqueous solutions (5, 10, 20, 30 wt%) [5C/H, 10C/H, 20C/H, 30C/H] and placed in an ultrasonic bath for removal of gases (1 h). Next, degassing mixture was allowed to shake (130 rpm) for 24 h at room temperature to ensure that saccharose could fill the outer and inner space between halloysite particles. After removing excessive saccharose solution, the solid was dried in 100 °C (24 h) and carbonized in a tubular furnace at 800 °C for 8 h with a heating rate of 5 °C/min under N_2_ atmosphere. Finally, the black product was milled and sieved. The obtained halloysite/carbon products were called 5C/H, 10C/H, 20C/H, and 30C/H.

### 2.3. Halloysite/Carbon Composite Characterization 

Textural structure of halloysite/carbon composites was characterized using the methods of low-temperature nitrogen adsorption–desorption isotherms (−196 °C) on a volumetric adsorption analyzer ASAP 2020 by Micromeritics (Norcross, GA, USA) (Structural Research Laboratory of Jan Kochanowski University in Kielce). Before measurement, all of the samples were degassed at a temperature of 200 °C for 2 h. Specific surface area (S_BET_) of investigated carbon materials was determined with the Brunauer–Emmett–Teller (BET) method at a range of relative pressure from 0.05 to 0.2, considering the surface occupied by a single molecule of nitrogen in an adsorptive monolayer (cross-sectional area equals 0.162 nm^2^) [36,37]. Total pore volume (Vt) (the sum of micropore volume (V_mi_) and mesopores (V_me_)) was calculated from one point of nitrogen adsorption isotherm, corresponding to the relative pressure p/p_o_ equals 0.99 [37].

Images of halloysite/carbon composites were obtained by SEM–EDS measurements carried out on a Tescan Vega3 microscope with LaB6 as electron source. Accelerating voltage was set to 5 kV in order to avoid excessive sample charring. Samples were put on standard SEM studs covered with an adhesive copper tape. In order to carry out elemental analysis, an Oxford Instruments x-act EDS detector was used. Additional high resolution SEM images were recorded after sputtering gold on the sample and using 30 kV accelerating voltage.

Infrared spectra were recorded using a Perkin-Elmer Spectrum 400 FT-IR/FT-NIR spectrometer with a smart endurance single bounce diamond, attenuated total reflection (ATR) cell. Spectra in the 4000–650 cm^−1^ range were obtained by the co-addition of 500 scans with a resolution of 4 cm^−1^. Before the measurements, all samples were dried and powdered in an agate mortar. 

Mineralogical compositions and sample structure were determined by the XRD analysis. X-ray diffraction patterns were collected using a Panalytical Empyrean diffractometer, equipped with a copper-based anode (Cu-K_α_ LFF HR, λ = 0.154059 nm). The instrument settings were 40 mA and 45 kV and the step of scans and counting time were 0.02° and 2 s per step, respectively.

Total carbon (TC) was quantified using an ELTRA CHS580 elemental analyzer (Neuss, Germany) (AGH). Total carbon was analyzed by placing approximately 0.250 g of dried, ground, and homogenized sample into a clean, carbon-free combustion boat. The sample boat was loaded into the furnace at 1350 °C. The combustion of the sample was performed in an oxygen atmosphere. Carbon content was determined on the basis of the amount of CO_2_ formed and the mass of the sample used for the analysis. An IR detector was used to measure CO_2_.

Functional groups on the surface 5C/H, 10C/H, 20C/H, and 30C/H composites were identified using Boehm’s titration method [38]. The Boehm methodology is based on quantifying different functional groups from titrations with bases of different strength: NaOH solutions were used to titrate the sum of carboxylic acids, phenols, and lactones; Na_2_CO_3_ and NaHCO_3_ were used to titrate carboxylic acids and lactones. Basic groups were quantified by titration with HCl as a total sum. The procedure was given a detailed description in [39]. The pH value at point of zero charge (pH_PZC_) was determined by mass titration. In particular, 0.15 g of halloysite/carbon sample was placed in a beaker with aliquots of 50 mL of 0.1 M NaCl added. The samples were stored under agitation (150 rpm) and constant temperature (25 °C) for 3 h. After that, the pH of each solution was regulated by adding 0.1 M HCl and 0.01 M NaOH measured in the range of 1 to 12 [40]. 

### 2.4. Adsorption Measurements

Batch adsorption experiments were carried out in a 100 cm^3^ Erlenmayer’s flask containing a proper amount of adsorbent and 30 mL ultrapure water from the Milli Pore system. Next, 30 ml of the investigated adsorbate solution was added and the flask was put into the incubator for a defined period of time: 10, 15, 30, 45, 60, 120, 240, 300, 360, and 1440 minutes. The measurements were performed at 25 °C and a mixing rate of 150 rpm. After that, adsorbent was separated from the solution with cup-type centrifuge. Concentrations of ketoprofen, diclofenac, and naproxen solutions before and after the adsorption were determined with the spectrophotometric method, using a UV Shimadzu UV-1800 spectrophotometer. The wavelengths used to determine the studied adsorbate concentrations were as follows: 260.5 nm (ketoprofen), 275.5 nm (diclofenac), and 230.5 nm (naproxen). 

The removal efficiency (R, %) and the amount of NSAIDs adsorbed at equilibrium (adsorption capacity, *q_e_*, mg/g) were calculated using Equations (1) and (2):

(1) $$R\;\left(\%\right)=\frac{C_{0}-C_{e}}{C_{0}}\times 100$$

(2) $$q_{e}=\frac{{(C}_{0}-C_{e})V}{m}$$

where *C*_0_ and *C_e_* (mg/L) are the initial and equilibrium concentrations of adsorbate solutions, *V* (L) is the volume of the adsorbate solution, and *m* (g) is the mass of adsorbent.

Adsorbate concentration as a function of the adsorbed amount at equilibrium is an important relation in determination of attraction nature for a given adsorption system. In addition, the analysis of equilibrium data is useful for designing the adsorption unit. We checked the fit of the experimental data to the following adsorption models: Langmuir (one-center and multi-center) [41], Freundlich [42], Temkin [43], and Dubinin–Radushkevich [10]; together with three-parameter isotherms like Sips [44]. The names and equation forms of common adsorption models are collected in Table 3.

## 3. Results and Discussion

### 3.1. Computational Calculation

#### 3.1.1. Computational Methodology

The molecules and their corresponding ions were modeled by DFT (Density Functional Theory) calculations using a commercial Scigress program (version FJ 2.7). The structures of the studied compounds were subject to geometry optimization using the B88-LYP GGA functional and the DZVP basis [45,46].

##### Calculations

**Figure 1:**
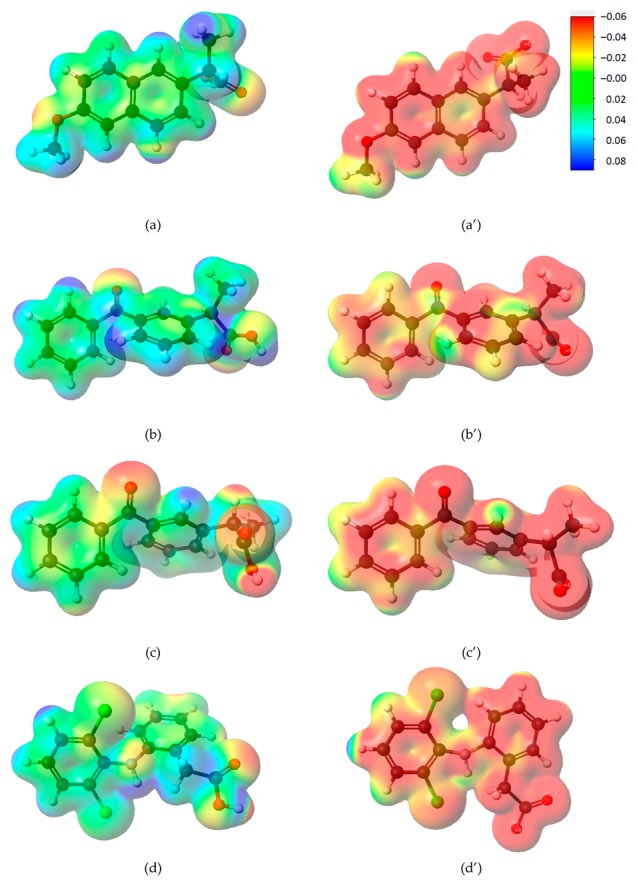
Electrostatic potential energy maps for (**a**) (*S*)-naproxen, (**a’**) anion of (*S*)-naproxen, (**b**) (*R*)-ketoprofen, (**b’**) anion of (*R*)-ketoprofen, (**c**) (*S*)-ketoprofen, (**c’**) anion of (*S*)-ketoprofen, (**d**) diclofenac, (**d’**) anion of diclofenac. Particular atoms have been marked with colors: Carbon—black; hydrogen—white; nitrogen—blue; oxygen—red; chlorine—green. Blue surface indicates positive value of electrostatic potential (localized positive charge), and the red one, negative value (localized negative charge); the surface legend is in upper right corner. Electron density at the surface is 0.01 *e*/*A*^3^.

Electrostatic potential energy maps for naproxen, ketoprofen, diclofenac, and their anions are presented in Figure 1. In dependence on pH, the studied molecules exist in solutions in an undissociated form or as anions as indicated by the calculated degree of dissociation values collected in Table 4. Mainly undissociated molecules occur in highly acidic environments. As pH increases, more dissociated molecules appear. In an alkaline environment, 43% and 34% of the naproxen and ketoprofen molecules are dissociated, respectively. Therefore, it is advisable to include the electronic description of the molecules to explain its interaction with the adsorbent surface. The used B88-LYP GGA functional does not fully include long-range-type electrostatic effects [47,48,49,50,51], but assuming that van der Walls forces will work on all molecules, the absolute values of partial molecular charges allow comparison of their electron distributions and capture general trends. Considering the interaction of different molecules and anions with the same adsorbent surface, electrostatic potential energy maps for adsorbates were prepared (Figure 1). These visualizations allow one to see different electronic density distribution of adsorbates and its anions. To gain deeper insight into divergent adsorption of the studied compounds, energy values of HOMO and LUMO orbitals were used to calculate the HOMO–LUMO energy gap, $\Delta E_{HL}$, and global indexes like chemical potential, *μ*, chemical hardness (*η*), and electrophilicity index, *ω* [52,53]:

(3) $$\Delta E_{HL}=\left|E_{H}-E_{L}\right|$$

(4) $$\mu =-\frac{E_{H}+E_{L}}{2}$$

(5) $$\eta =\frac{E_{L}-E_{H}}{2}$$

(6) $$\omega =\frac{\mu^{2}}{2\eta }$$

where *E_H_* and *E_L_* are the HOMO and LUMO orbital energies, respectively.

Theoretical values of electronic descriptors of all adsorbates are given in Table 5. For pH equal to 1, one should compare electronic properties of undissociated molecules, because they occur mainly in strongly acidic solutions (Table 4). The lower value of energy gap had ketoprofen (2.96 eV), which indicates its greatest reactivity. Ketoprofen was also the most electrophilic with *μ* equal to 4.06 eV and 4.10 eV and *ω* equal to 5.57 eV and 5.68 eV for isomer *S* and *R*, respectively. However, these high values of electrophilicity could cause a decrease in its reactivity, because the adsorbent surface at pH = 1 is positively charged. Higher values of the chemical hardness for undissociated molecules than for its anions show that electron densities of undissociated adsorbates were harder to modify. Nevertheless, the values of *η* for undissociated molecule are not very high, and that is why these adsorbates can react by electrostatic interactions. 

As the pH increases, the number of dissociated molecules increases as well, which is shown in Table 4. At pH equals 4 and 6, a large amount of anions means that there are more and more electron donors and, at the same time, there is still a positive charge on the surface of the adsorbent. Anions of all the studied molecules have low hardness values, which means that they are “soft” molecules and their electron densities can be easily changed. This causes that the predicted adsorption mechanism is based on electron sharing between acid surface and adsorbate anions.

In an alkaline environment, there is a negative charge on the adsorbent surface. That is why strongly electrophilic molecules interact with this surface. The highest values are for (*R*)- and (*S*)-ketoprofen, next (*S*)-naproxen. Diclofenac appears to be the least adsorbed in alkaline pH. Electrostatic potential energy maps (Figure 1) show that there is much more positive charge on adsorbate surface, which allows the molecules to interact with the surface in various configurations. 

### 3.2. Characterization of Adsorbents

**Figure 2:**
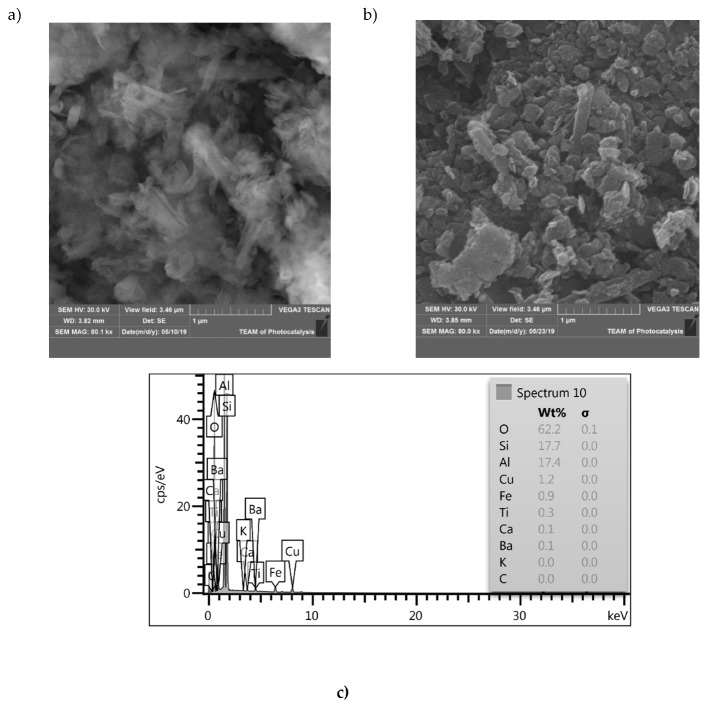

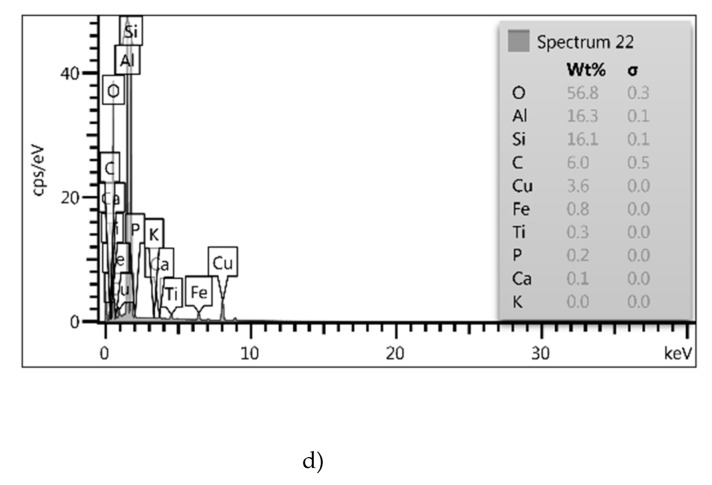
SEM images of H (**a**), 30C/H (**b**), and EDS spectra of H (**c**) and 30C/H (**d**).

The morphologies of H and 30C/H samples were observed by means of SEM and EDX, as shown in Figure 2. It can be seen that sample H consists of tubular, blocky, and platy particles (Figure 2a). Tube lengths were up to a few hundred nanometers and were arranged in various directions. In the case of the 30C/H sample, one can see numerous carbon particles with irregular shapes deposited on the halloysite surface (Figure 2b). Using EDS analysis (Figure 2c,d), the presence of carbon in the synthesized composite 30C/H was confirmed. Carbon content in the 30C/H sample was equal to 6 wt%, and no carbon was found in the H sample.

**Figure 3:**
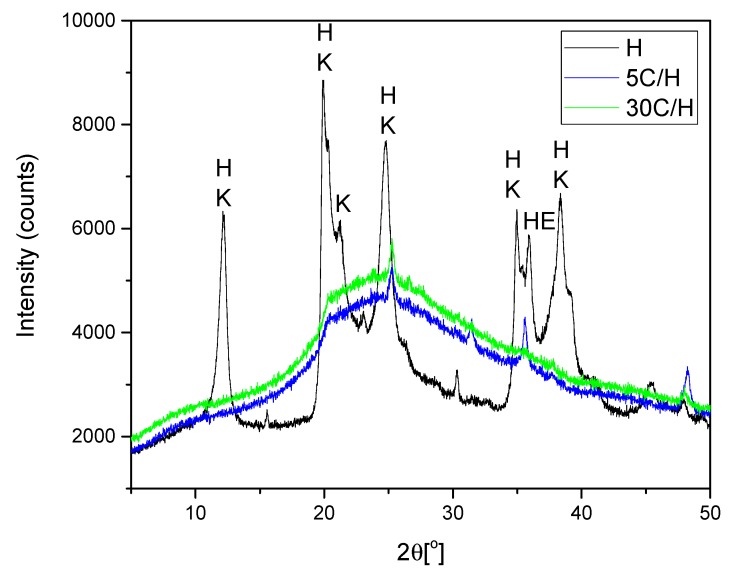
Diffraction patterns of samples: H, 5C/H, and 30C/H (see text for description). In the diffractograms, the peaks of the following minerals were identified: H—halloysite; K—kaolinite; He—hematite.

Diffraction patterns of H, 5C/H, and 30C/H samples are compared in Figure 3 (the diffractograms of 10C/H and 20C/H are very similar to diffraction patterns of 5C/H and 30C/H samples). The main reflections of the halloysite, kaolinite, and hematite are identified for the H sample. XRD patterns of 5C/H and 30C/H samples confirm that amorphous carbon covered the halloysite surface. These diffractograms are very similar to diffraction patterns of amorphous carbons produced using glucose, lactose, and saccharose, as mentioned by Myronyuk et al. [21] with the main reflection at 2θ = 22° and very weak at 43°. The reflection at 25° and 48° can be assigned to the crystalline structure of formed activated carbon. This connected to alignment of disordered graphitic carbon layers, which form the crystalline turbostatic structure [54]. The XRD line at 2θ = 36° for 5C/H corresponds to hematite [35], which is the rest of the iron not totally removed during the preparation.

**Figure 4:**
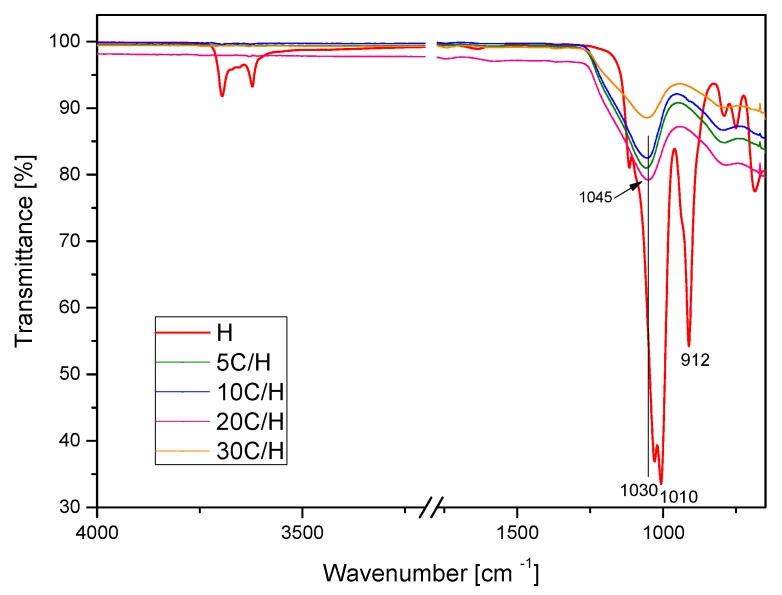
FTIR spectra of H, 5C/H, 10C/H, 20C/H, and 30C/H samples in the 4000–650 cm^−1^ region.

ATR FT-IR spectra of halloysite (H) and halloysite/carbon composites (5C/H, 10C/H, 20C/H, and 30C/H) in the 4000–650 cm^−1^ region are presented in Figure 4. The FT-IR spectrum of the H sample shows characteristic bands for the kaolin-group minerals: In the 3700–3600 cm^−1^ region, the vibration of the OH group, and in the 1750–650 cm^−1^ region, bands assigned to apical Si–O (1107 cm^−1^) and to perpendicular stretching vibrations of Si–O–Si (1030 and 691 cm^−1^) [35,55,56]. The bands in the range of 3700–3600 cm^−^^1^, corresponding to stretching oscillations of the O–H, were not observed for all halloysite/carbon composites. Band intensity in the 1750–650 cm^−1^ range significantly decreased. The band at 1030 cm^−1^ shifted to the position at 1045 cm^−1^. This shifting suggests the change on the outer surfaces of halloysite (silica tetrahedron sheet), probably caused the presence of carbon on this surface. 

**Figure 5:**
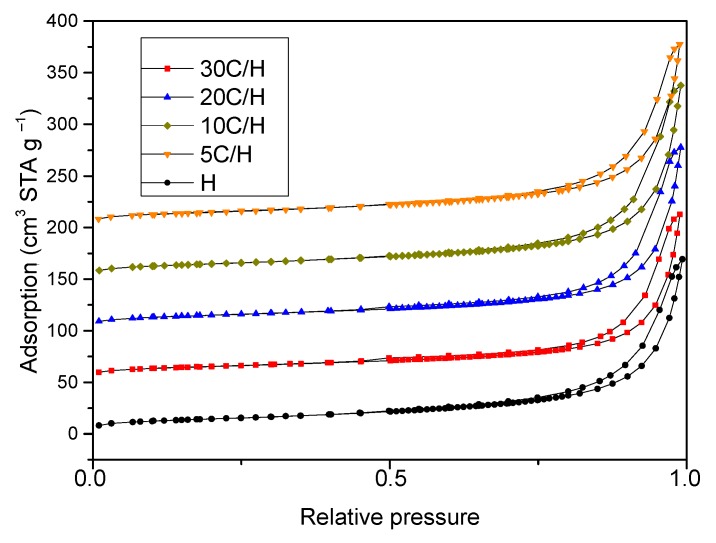
Nitrogen adsorption–desorption isotherms for the H sample and halloysite/carbon composites.

Experimental adsorption–desorption N_2_ isotherms of the H sample and halloysite/carbon composites (Figure 5) are type IV according to the IUPAC classification [57], which indicates a mesoporous character of these materials. Structural parameters calculated from adsorption isotherms are presented in Table 6. BET specific surface area (S_BET_) was higher for the H sample (73 m^2^/g) in comparison to halloysite/carbon composites. S_BET_ for all composites was comparable and equals 53 m^2^/g for the 5C/H sample up to 55 m^2^/g for the 30C/H sample. Carbon deposition on the halloysite surface did not significantly affect the total pore volume. The volume of micropores increased for 20C/H and 30C/H samples versus the H sample.

Analysis of total carbon showed that halloysite/carbon composites contained various amounts of carbon: Sample 5C/H—2.2 wt%; sample 10C/H—2.9 wt%; sample 20C/H–4.6 wt%; sample 30C/H—6.7 wt%.

In order to investigate the influence of functional groups present on the carbon surface on the adsorption of NSAIDs, Boehm analysis was carried out for the 30C/H sample (Table 7). The amount of acid groups was considerably higher than the basic groups on the surface of the 30C/H sample. Carboxyl, carbonyl, phenolic, and lactone groups were identified on the adsorbent surface. The point of zero change (pH_pzc_) of 30C/H equaled 6.56.

### 3.3. Adsorption Study

**Figure 6:**
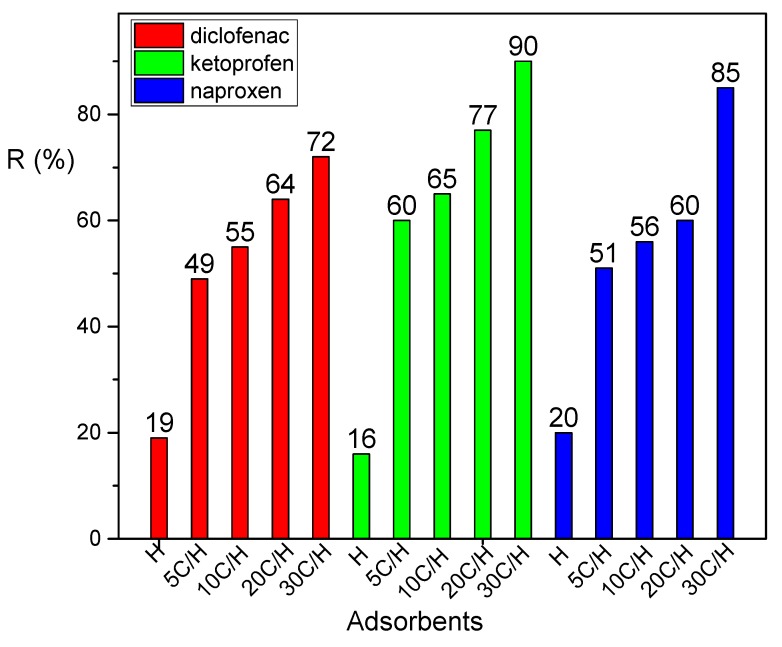
Removal efficiency of diclofenac, ketoprofen, and naproxen for halloysite (H) and halloysite/carbon adsorbents (concentration of adsorbate solutions—50 mg/dm^3^; mass of adsorbent—0.5 g, temp. 25 °C).

Removal efficiencies of diclofenac, ketoprofen, and naproxen from aqueous solution on adsorbents H, 5C/H, 10C/H, 20C/H, and 30C/H are presented in Figure 6. The results show that halloysite/carbon composites adsorb diclofenac, ketoprofen, and naproxen better than halloysite (H). Removal efficiency increased with the increase of saccharose concentration for all studied NSAIDs. The best results were obtained for adsorption on 30C/H adsorbent for all studied adsorbates. The values of removal efficiency for 30C/H adsorbent increased in the following order: Diclofenac (72%) < naproxen (85%) < ketoprofen (90%). 

#### 3.3.1. Effect of Adsorbent Dose and pH on NSAID Adsorption

**Figure 7:**
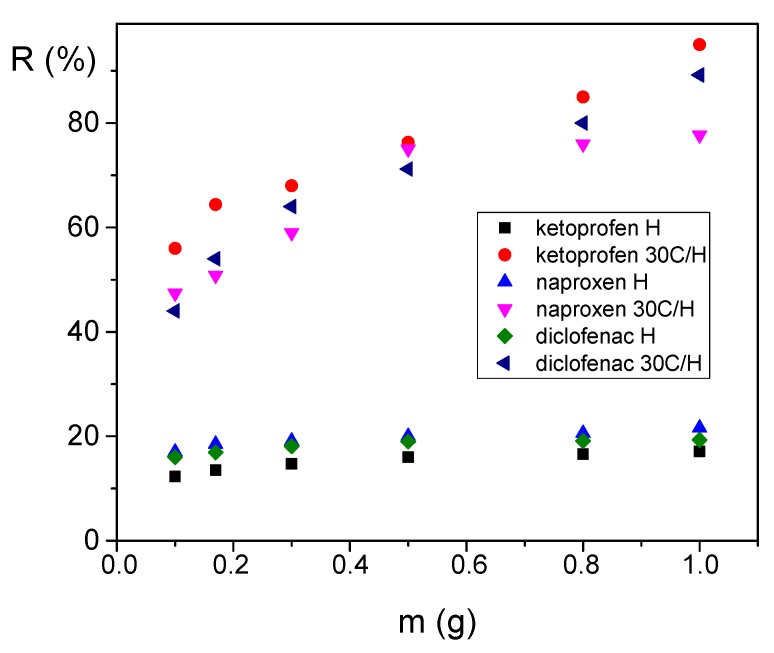
Dependence of removal efficiency of diclofenac, ketoprofen, and naproxen on mass of adsorbents H and 30C/H (*C*_0_ = 50 mg/dm^3^, contact time 8 h).

The effect of adsorbent dose (H and 30C/H composite) on the adsorption of NSAIDs studied was investigated. Solutions of ketoprofen, naproxen, and diclofenac at a concentration of 50 mg/dm^3^ and an adsorbent dose from 0.1 to 1.0 g were used. Results are presented in Figure 7. Removal efficiency of all used adsorbates was higher for the adsorbent 30C/H compared to the H adsorbent, and increased together with the rise of the adsorbent dose, reaching a maximum value for 1.0 g of the 30C/H adsorbent.

**Figure 8:**
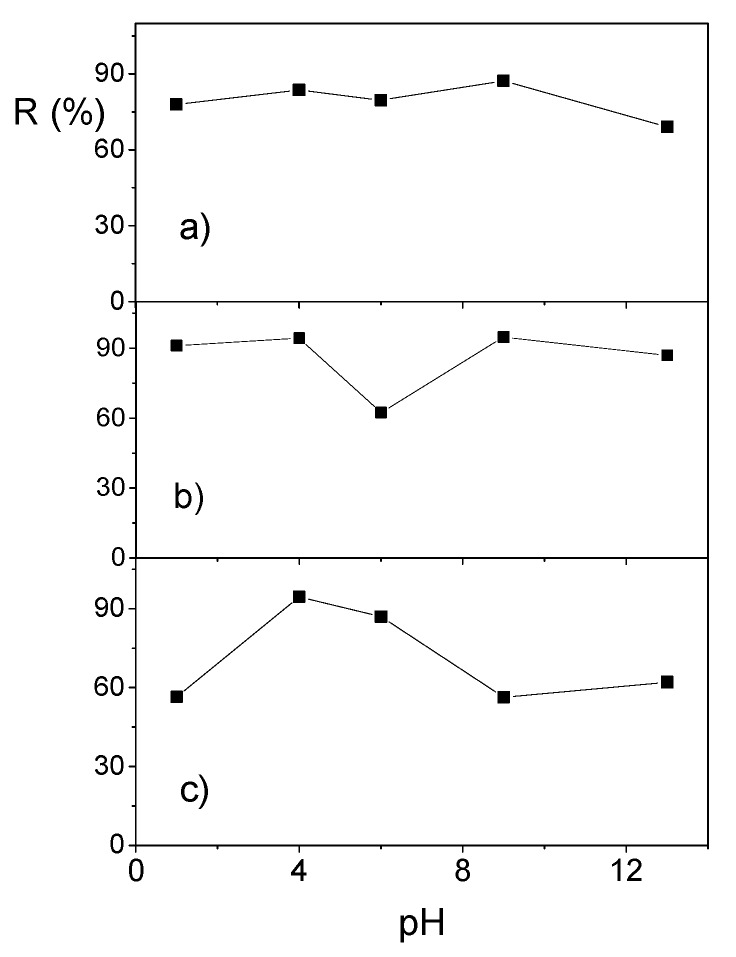
Dependence of removal efficiency of ketoprofen (**a**), naproxen (**b**), and diclofenac (**c**) on pH of solution for the 30C/H adsorbent (*C*_0_ = 50 mg/dm^3^, contact time 8 h).

The adsorption of NSAIDs from an aqueous solution on carbonaceous adsorbents was mainly due to the interactions between the functional groups in the NSAID molecule and the functional groups on the adsorbent surface [58]. Ketoprofen, diclofenac, and naproxen, weak organic acids, coexist in both ionized and non-ionized forms in aqueous solution. The distribution of these forms is dependent on the pH of solution, and their interaction with adsorbent surface may or may not favor the adsorption process. The concentration of these conjugates depends on the solution pH and pK_a_. Based on pK_a_ values of adsorbates (see Table 4), and using the Henderson–Hasselbalch equation, the amount of acidic and basic forms of adsorbate molecules was calculated. In distilled water (pH = 6), the ionization degree was 34.22% for ketoprofen and 42.91% for naproxen. Diclofenac exists only as anion, because we used its sodium salt in adsorption experiments. The value of pH_PZC_ represents the pH of the solution, at which the net surface charge was neutral. At a solution pH lower than the pH_PZC_, total surface charge will be, on average, positive, whereas at a higher solution pH, they will be negative because of the deprotonation of functional groups [58]. pH of adsorbate solution had a significant influence on NSAID adsorption on halloysite/carbon composites, because it was one of the key factors that controlled the adsorption process of organic weak electrolytes on carbon materials, especially in the case of the electrostatic interactions between the adsorbent and the adsorbate. The effect of solution pH on removal efficiency (R%) of ketoprofen, diclofenac, and naproxen on 30C/H adsorbent is shown in Figure 8. Removal efficiency of ketoprofen practically did not change in the range of pH 1–9, only for a pH of 13 was it slightly decreased. For naproxen, the value of R was the highest in the ranges pf pH 1–4 and 9–14, and clearly lower for pH 6. The effect of pH on diclofenac adsorption was definitely larger. The highest values of R were obtained for the range of pH 4–6, whereas the lowest for pH equal to 1, 9, and 13. In the range of pH 4–13, naproxen and ketoprofen were dissociated from 20% to about 43%, and the neutral, as well as dissociation form molecules, existed in solution. Both forms may interact with a positive- or negative-charged adsorption surface, so the effect of pH on their removal efficiency was lower than for diclofenac. At basic pH, the uptake for diclofenac was lower, because of electrostatic repulsions between the negative surface charge and the diclofenac anions and between diclofenac anions in solution. At a solution pH between 4 and 6 (lower than the pH_PZC_ for 30C/H adsorbent), the total surface charge was, on average, positive, and the electrostatic interaction was stronger between diclofenac anions and adsorbent surface, resulting in higher uptake. 

Adsorption mechanism concerning ketoprofen, naproxen, and diclofenac from dilute aqueous solutions on halloysite/carbon material includes electrostatic and non-electrostatic interactions. Electrostatic interactions appeared when adsorbate molecule electrolytes were ionized in the experimental conditions used. Non-electrostatic interactions were due to dispersion and hydrophobic interactions between non-dissociated adsorbate molecules and the adsorbent surface containing mainly oxygen acidic groups.

Electrostatic interactions do not completely explain the mechanism of adsorption of NSAIDs on the 30C/H adsorbent. One of the features of the adsorption mechanism is that aromatic compounds on carbon materials may include the π–π dispersion interaction, the possibility of H-bond formation with surface oxygen groups, such as carboxyl or carbonyl groups or donor–acceptor electron complex formation [59]. These interactions between NSAID molecules or their anions and adsorbent surfaces are also possible, and can further complicate adsorption mechanism.

The following conditions were selected as optimal conditions for NSAID adsorption on studied adsorbents: Reaction temperature 25 °C, pH 6, and catalyst dosage 0.5 g.

#### 3.3.2. Kinetic Models.

**Figure 9:**
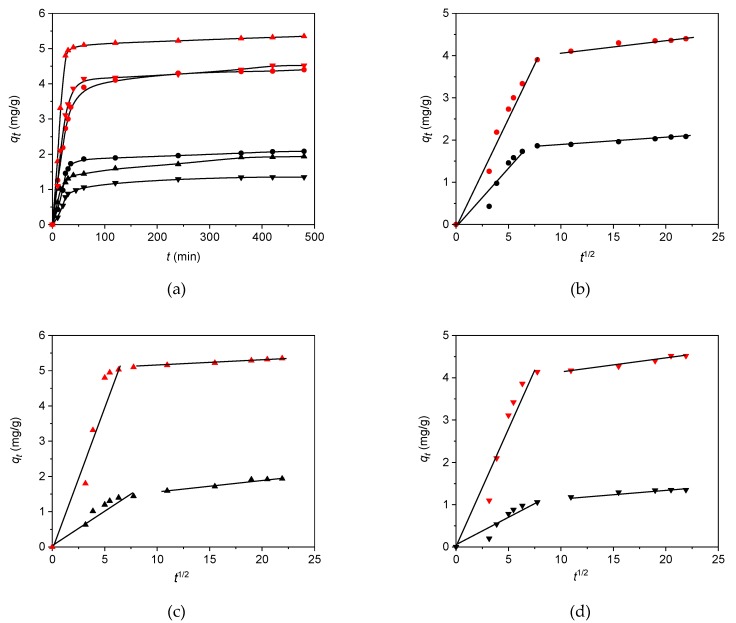
Kinetic adsorption curves (**a**), *C*_0_ 50 mg/dm^3^ and the intra-particle diffusion (**b**–**d**) of diclofenac, ketoprofen, and naproxen on H and 30/CH adsorbents. ●—diclofenac; ▲—ketoprofen; ▼—naproxen; black symbol, H; red symbol, 30/CH.

The pseudo-first-order kinetic model [59], pseudo-second-order kinetic model [60], and intra-particle diffusion model [61] (Table 3) were investigated for the adsorption of ketoprofen, naproxen, and diclofenac on H and 30C/H adsorbents. Adsorption kinetics for ketoprofen, naproxen, and diclofenac on H and 30C/H adsorbents are presented in Figure 9a. Adsorption equilibrium was obtained after 80 min for all of the studied NSAIDs. The pseudo-first-order and pseudo-second-order rate constants, k_1_ and k_2_, and correlation coefficients (R^2^) are collected in Table 8. The values of correlation coefficients obtained for the pseudo-first-order kinetic and pseudo-second-order kinetic models clearly indicate that adsorption of the studied compounds on H and 30C/H adsorbents obey the pseudo-second-order kinetic model, suggesting that the electrochemical interactions play an important role in the adsorption process. The values of pseudo-second-order rate constant, k_2_, were greater for all adsorbates for 30C/H adsorbent in comparison to the H adsorbent and decreased in the following order: Ketoprofen > naproxen > diclofenac for both adsorbents. 

The Weber–Morris diffusion model was used in order to investigate adsorption mechanism of ketoprofen, naproxen, and diclofenac on the H and 30C/H adsorbents. The diffusion model is presented by the equation given in Table 3.

The values of k_d1_, k_d2_ and c_1_, c_2_ determined from the slopes and intercepts of the first and second linear part of graph (Figure 9b–d) are given in Table 9. Constant values k_d1_ decreased in the following order: Naproxen > ketoprofen > diclofenac for H and ketoprofen > naproxen > diclofenac for 30C/H adsorbents. For both adsorbents, the rate of diffusion was the smallest for diclofenac, i.e., for the adsorbate of the highest molecular weight.

The dependency *q_t_* vs. *t*_1/2_ multi-linear plot (broken line on the graph) indicates that in the adsorption process, several steps are involved. The first section on the graph corresponds to the faster step, attributed to the diffusion of adsorbate molecules to adsorbent outer surface while the second part of the graph corresponds to slower adsorption, where intra-particle diffusion is a controlling step of the whole adsorption process [62].

#### 3.3.3. Adsorption Isotherms

**Figure 10:**
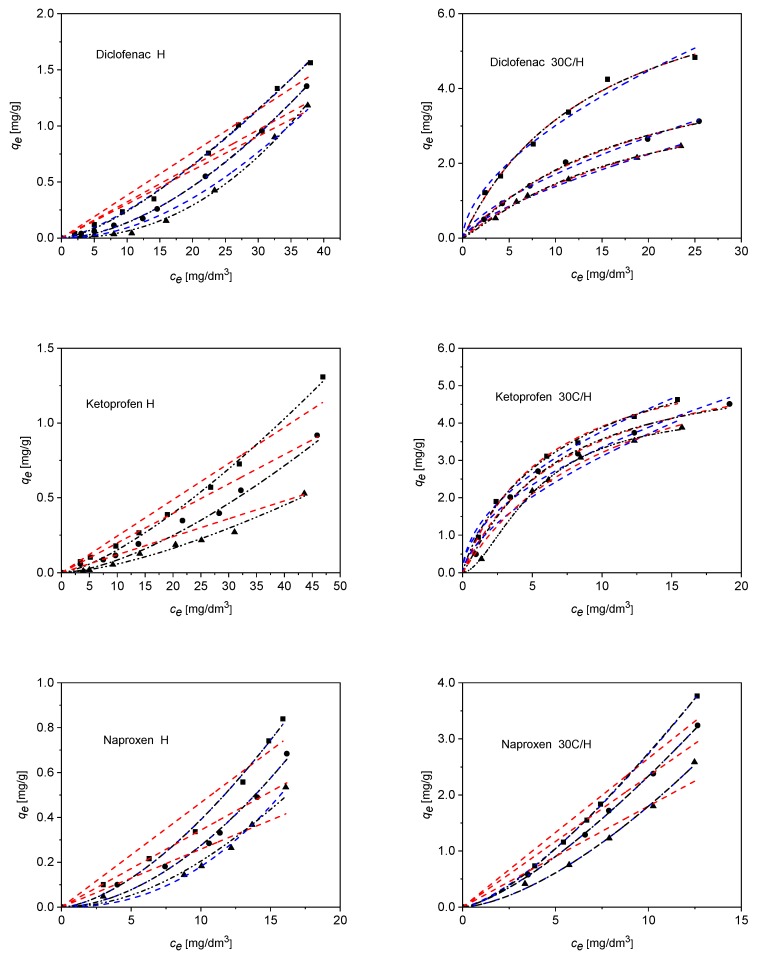
Calculation of adsorption equilibrium constants for diclofenac, ketoprofen, and naproxen on H and 30C/H adsorbents employing the experimental data. The lines represent the curve obtained by the application of the Freundlich equation (blue dash), Langmuir one-center equation (red dash), and Langmuir multi-center equation (black dash dot dot), with respect to the adsorption data as adjusted by the least squares method. Temperature: ■—298 K; ●—303 K; ▲—313 K.

The results show a complete lack of correlation of experimental data for following models: Temkin, Dubinin–Radushkevich, and Sips. In the interpretation of the experimental data, three different models were used: the Freundlich, the Langmuir one active center without dissociation (one-center), and the Langmuir adsorption model on multiple active centers without dissociation (multi-center) [62,63]. Fitting experimental data to the isotherm models given in Table 3 was performed using non-linear regression (Levenberg–Marquardt least squares method with the Origin Microcal software). The results of the fitting as regards experimental data to the Freundlich and Langmuir (one-center and multi-center) isotherm models are shown in Figure 10. The Freundlich and Langmuir (one-center and multi-center) equation parameters, as well as correlation coefficients R^2^ for the adsorption of ketoprofen, diclofenac, and naproxen on H and 30C/H adsorbents, are collected in Table 10, Table 11 and Table 12. The highest values of correlation coefficients for ketoprofen, diclofenac, and naproxen adsorption on H and 30C/H adsorbents were obtained for the Langmuir adsorption model on multiple active centers without dissociation (multi-center) model applied to fit experimental data. Adsorption constant values were lower for adsorbent H than for adsorbent 30C/H, and decreased for both adsorbents in the following order: Ketoprofen > diclofenac > naproxen. An increase in temperature caused a decrease in the these constant values, indicating the exothermic nature of the adsorption process. The *n* values were fractional for all adsorbates, pointing out the adsorption mechanisms of the NSAID molecules with a different number of adsorptive centers on H and 30C/H surfaces. The shape of adsorption isotherms is similar in the case of ketoprofen and naproxen, while it is different in the case of diclofenac, suggesting differences in the mechanism of adsorption of these compounds on 30C/H adsorbent.

## 4. Conclusions

The adsorption of ketoprofen, naproxen, and diclofenac from an aqueous solution on halloysite and new halloysite/carbon nanocomposite was studied. Removal efficiency of all the studied NSAIDs for halloysite/carbon nanocomposites was significantly higher than for non-modified halloysite. Adsorption kinetics for ketoprofen, naproxen, and diclofenac on the halloysite and halloysite/carbon nanocomposites can be described with the pseudo-second-order kinetic model. The adsorption process of ketoprofen, naproxen, and diclofenac on halloysite and halloysite/carbon adsorbents proceeded in compliance with the Langmuir (multi-center) adsorption model (Langmuir adsorption model on multiple active centers without dissociation). 

Theoretical values of electronic descriptors showed the greatest reactivity of ketoprofen, lower for naproxen, and the lowest for diclofenac, which is in agreement with the order of changes in rate constants and adsorption constants for all studied adsorbates.

Halloysite as a cost-saving, environmentally-friendly nano-carrier combined with carbon material may be a probable suitable adsorbent of all the studied pharmaceuticals for large scale application.

## Figures and Tables

**Table 1:** Adsorbents used for ketoprofen, naproxen, and diclofenac removal from aqueous solutions.

Adsorbent	Adsorbate	Adsorption Conditions	Ref.
bentonite, kaolinite, montmorillonite, vermiculite, acid-modified montmorillonite, carbonaceous–mineral nanocomposites: The initial material—commercial bentonite or montmorillonite intercalated with aluminum polycation	diclofenac ketoprofen	0.2 g/10 mL, 24 h, 20 °C, pH 3.1–8.4	[7]
Zeolite-modified cetyltrimethylammonium bromide (CTAB) or cetyltrimethylammonium chloride (CTAC)	diclofenac	0.25 g/10 mL, 24 h, 32, 42, and 52 °C, pH 2−11	[8]
Activated carbon obtained from olive waste (activator H_3_PO_4_)	naproxen ketoprofen diclofenac	0.3−1.5 g/L, 26 h, 25 °C, pH 4.1	[10]
Hydrochar from orange peels	diclofenac	0.015 g/30 mL, 25 °C, neutral pH	[12]
N-/O-doped porous carbons derived from MOF (ZIF-8 —zeolitic imidazolate framework-8)	diclofenac	4 mg/50 mL, 12 h, 25 °C, pH 1−11	[14]

**Table 2:** Chemical structures and selected properties of the adsorbates [34].

Compound	Ketoprofen	Diclofenac	Naproxen
**Molecular structure**	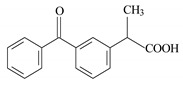	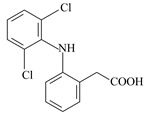	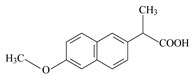
**IUPAC name**	2-(3-benzoylphenyl) propanoic acid	sodium;2-[2-(2,6-dichloroanilino)phenyl] acetate	(+)-(*S*)-2-(6-methoxy-naphthalen-2-yl)propanoic acid
**Chemical formula**	C_16_H_14_O_3_	C_14_H_11_C_l2_NNaO_2_	C_14_H_14_O_3_
**Molecular weight**	254.285 g/mol	296.148 g/mol	230.26 g/mol
**Water solubility**	51 mg/L (25 °C)	33 mg/L (25 °C)	15.9 mg/L (25 °C)
pKa *	4.45	4.15	4.15
λ (nm) **	260	275	230

* K_a_—dissociation constant at 20 °C. ** absorption wavelength at which spectrophotometric measurements were made for adsorbates.

**Table 3:** The names and equation forms of common adsorption models.

**Adsorption isotherms**		
Freundlich	$q_{e}=K_{F}C_{e}^{1/n}$	*K_F_*—Freundlich constant for a heterogeneous adsorbent (mg^−1/n^ (dm^3^)^1/n^ g^−1^); 1/*n*—the heterogeneity factor
Langmuir (one-center)	$q_{e}=\frac{q_{m}K_{L}C_{e}}{{1+K}_{L}C_{e}}$	*C_e_*—equilibrium concentration of a solute in an aqueous solution (mg dm^−3^); *q_e_*—the amount of a solute adsorbed per gram of the adsorbent at equilibrium (mg g^−1^), *q_m_*—maximum monolayer coverage capacity (mg g^−1^); *K_L_*—Langmuir isotherm constant (dm^3^ g^−1^)
Langmuir (multi-center)	$q_{e}=\frac{q_{m}{\left(K_{LF}C_{e}\right)}^{1/n}}{1+{\left(K_{LF}C_{e}\right)}^{1/n}}$	*C_e_*—equilibrium concentration of a solute in an aqueous solution (mg dm^−3^); *q_e_*—the amount of a solute adsorbed per gram of the adsorbent at equilibrium (mg g^−1^), *q_m_*—maximum monolayer coverage capacity (mg g^−1^); *K_LF_*—Langmuir–Freundlich isotherm constant (dm^3^ g^−1^), *n*—adsorption model index
**Kinetics models**		
Pseudo-first-order	$ln\left(q_{e}{-q}_{t}\right)=lnq_{e}-k_{1}t$	*k*_1_—pseudo-first-order rate constants (min^−1^); *t*—time of contact between the adsorbent and adsorbate (min); *q_e_*—amount of adsorbate at equilibrium (mg g^−1^); *q_t_*—amount of adsorbate at time *t* (mg g^−1^)
Pseudo-second-order	$\frac{t}{q_{t}}=\frac{1}{k_{2}q_{e}^{2}}+\frac{t}{q_{e}}$	*k*_2_—pseudo-second-order rate constants (g mg^−1^ min^−1^)
Intraparticle diffusion	$q_{t}=k_{d}t^{1/2}+c$	*k_id_*—intra-particle diffusion rate constant (mg g^−1^ min^−1/2^); *c*—intercept, which represents the thickness of the boundary layer (mg g^−1^)

**Table 4:** The degree of dissociation values for (*S*)-naproxen, (*R*)-ketoprofen, and (*S*)-ketoprofen for different pH.

Adsorbate	pH	α [%]
(*S*)-naproxen	1	0.07
	4	30.00
	6	42.91
	9	43.08
	13	43.08
(*R*)-ketoprofen (*S*)-ketoprofen	1	0.04
	4	20.25
	6	34.22
	9	34.42
	13	34.42

**Table 5:** The values of electronic descriptors of adsorbates ($\Delta E_{HL}$—the HOMO–LUMO energy gap; *μ*—chemical potential; *η*—chemical hardness; *ω*—electrophilicity index).

Chemical Sample	*E_H_* [eV]	*E_L_* [eV]	*E_HL_* [eV]	*μ* [eV]	*η* [eV]	*ω* [eV]
(*S*)-Naproxen	−4.96	−1.75	3.21	3.36	1.61	3.51
Ion of (*S*)-Naproxen	0.06	1.21	1.15	−0.64	0.58	0.35
(*R*)-Ketoprofen	−5.58	−2.62	2.96	4.10	1.48	5.68
Ion of (*R*)-Ketoprofen	−0.25	−0.01	0.24	0.13	0.12	0.07
(*S*)-Ketoprofen	−5.54	−2.58	2.96	4.06	1.48	5.57
Ion of (*S*)-Ketoprofen	−0.28	0.00	0.28	0.14	0.14	0.07
Diclofenac	−4.96	−1.62	3.34	3.29	1.67	3.24
Ion of Diclofenac	−0.21	0.80	1.01	−0.30	0.51	0.09

**Table 6:** Structural parameters of the studied materials.

Adsorbents	S_BET_ m^2^/g	V cm^3^/g	V_me_ cm^3^/g	V_mi_ cm^3^/g	Mesoporosity %
H	73	0.24	0.23	0.0019	99.5
5C/H	53	0.25	0.25	0.0015	99
10C/H	53	0.26	0.26	0.0015	99
20C/H	54	0.25	0.24	0.0032	98
30C/H	55	0.22	0.22	0.0048	98

S_BET_—specific surface area; V—single-point total pore volume calculated at p/p_o_ = 0.99; V_me_—mesopore volume calculated by subtracting V_mi_ from V; V_mi_—volume of micropores obtained by α_s_-method; Mesoporosity—the percentage of the mesopore volume in relation to the total pore volume.

**Table 7:** Functional groups available on the studied materials.

Adsorbents	Total Basic Groups (mmol g^−1^)	Total Acidic Groups (mmol g^−1^)	Total Carboxyl Groups (mmol g^−1^)	Total Carbonyl Groups (mmol g^−1^)	Total Phenolic Groups (mmol g^−1^)	Total Lactone Groups (mmol g^−1^)
30C/H	2.9970	4.8522	0.5151	0.5434	2.6548	1.1389

**Table 8:** Kinetic parameters of ketoprofen, naproxen, and diclofenac adsorption on the studied adsorbents.

Adsorbate	Adsorbent	Pseudo-First-Order Kinetic Model		Pseudo-Second-Order Kinetic Model	
		k_1_ (min^−1^)	R^2^	k_2_ (g mg^−1^ min^−1^)	R^2^
**Diclofenac**	H	0.0119	0.6333	0,0246	0.9984
	30C/H	0.0128	0.5822	0.0497	0.9997
**Ketoprofen**	H	0.0115	0.9020	0.0371	0.9967
	30/H	0.0140	0.6123	0.0751	0.9999
**Naproxen**	H	0.0119	0.7598	0.0322	0.9983
	30C/H	0.0067	0.6535	0.0539	0.9992

**Table 9:** Intra-particle diffusion model parameters.

Adsorbate	Adsorbent	k_d1_ (mg g^−1^ min^−1/2^)	c_1_ (mg g^−1^)	R_1_^2^	k_d2_ (mg g^−1^ min^−1/2^)	c_2_ (mg g^−1^)	R_2_^2^
**Diclofenac**	H	0.0857	0.51	0.6682	0.0034	1.68	0.9184
	30C/W	0.2743	0.42	0.8930	0.0144	4.07	0.9635
**Ketoprofen**	H	0.1008	0.50	0.7174	0.0084	1.75	0,9281
	30C/W	0.5033	1.09	0.8951	0.0202	4.91	0.9997
**Naproxen**	H	0.1261	0.21	0.6720	0.0185	1.28	0.8677
	30C/H	0.4024	1.14	0.8523	0.0413	3.63	0.7856

**Table 10:** Freundlich and Langmuir (one-center and multi-center) equation parameters and correlation coefficients R^2^ for the adsorption of ketoprofen on H and 30C/H adsorbents.

Isotherms	Parameters	Ketoprofen					
		H			30C/H		
		Temperature [K]					
		298	303	313	298	303	313
Freundlich	*K_F_* (mg∙g^−1^) (dm^−3^∙mg^−1^)^1/n^		-	-	1.15423	1.02568	0.75201
	*n*		-	-	0.5148	0.51411	0.61633
	R^2^		-	-	0.9612	0.9678	0.9624
Langmuir (one-center)	*K_L_* (dm^3^∙mg^−1^)	0.0721	0.0345	0.0017	0.1603	0.1325	0.0916
	*q_m_* (mg∙g^−1^)	3.9	2.2	1.7	6.3	6.2	5.7
	R^2^	0.9684	0.9633	0.9664	0.9724	0.9873	0.9852
Langmuir (multi-center)	*K_L_* (dm^3^∙mg^−1^)^1/n^	0.0178	0.0142	0.0105	0.1463	0.1394	0.0593
	*q_m_* (mg∙g^−1^)	3.2	2.9	1.8	17.4	15.6	14.4
	*n*	1.58	1.55	1.49	0.86	1.12	1.07
	R^2^	0.9823	0.9817	0.9842	0.9913	0.9910	0.9872

**Table 11:** Langmuir, Freundlich, and Langmuir–Freundlich equation parameters and correlation coefficients R^2^ for the adsorption of naproxen on H and 30C/H adsorbents.

Isotherms	Parameters	Naproxen					
		H			30C/H		
		Temperature [K]					
		298	303	313	298	303	313
Freundlich	*K_F_* (mg∙g^−1^) (dm^−3^∙mg^−1^)^1/n^	0.0099	0.0041	0.0010	0.1130	0.0996	0.0511
	*n*	0.62	0.54	0.44	0.72	0.73	0.64
	R^2^	0.9822	0.9878	0.9848	0.9934	0.9967	0.9994
Langmuir (one-center)	*K_L_* (dm^3^∙mg^−1^)	0.0623	0.0583	0.0332	0.0531	0.0462	0.0363
	*q_m_* (mg∙g^−1^)	4.3	3.4	2.8	15.3	13.6	12.6
	R^2^	0.8678	0.8923	0.8843	0.,8523	0.8617	0.8733
Langmuir (multi-center)	*K_L_* (dm^3^∙mg^−1^)^1/n^	0.0046	0.0036	0.0022	0.0053	0.0018	0.0012
	*q_m_* (mg∙g^−1^)	4.6	2.3	1.5	16.4	13.2	12.4
	*n*	1.62	1.54	1.50	0.68	0.72	0.64
	R^2^	0.9922	0.9975	0.9952	0.9926	0.9991	0.9989

**Table 12:** Langmuir, Freundlich, and Langmuir–Freundlich equation parameters and correlation coefficients R^2^ for the adsorption of diclofenac on H and 30C/H adsorbents.

Isotherms	Parameters	Diclofenac					
		H			30C/H		
		Temperature [K]					
		298	303	313	298	303	313
Freundlich	*K_F_* (mg∙g^−1^) (dm^−3^∙mg^−1^)^1/n^	0.0099	0.0035	0.0021	0.8061	0.37306	0.27737
	*n*	0.72	0.58	0.45	0.57	0.66	0.69
	R^2^	0.8534	0.9022	0.8913	0.8645	0.8656	0.8722
Langmuir (one-center)	*K_L_* (dm^3^∙mg^−1^)	0.0843	0.0523	0.0257	0.0680	0.0453	0.0376
	*q_m_* (mg∙g^−1^)	3.8	2.0	1.7	6.8	5.7	5.2
	R^2^	0.9643	0.9726	0.9766	0.9892	0.9683	0.9768
Langmuir (multi-center)	*K_L_* (dm^3^∙mg^−1^)^1/n^	0.0092	0.0055	0.0017	0.0458	0.0387	0.0106
	*q_m_* (mg∙g^−1^)	3.8	1.4	0.9	17.8	14.9	14.0
	*n*	0.67	0.56	0.50	1.0	0.91	0.86
	R^2^	0.9903	0.9987	0.9992	0.9942	0.9975	0.9991

## References

[1] Cizmas L., Sharma V.K., Gray C.M., McDonald T.J. (2015). Pharmaceuticals and personal care products in waters: Occurrence, toxicity, and risk. Environ. Chem. Lett..

[2] Perrodin J.J., Pivot Y.C., Trepo D., Perraud M., Droguet J., Tissot-Guerraz F., Locher F. (2012). Identification and prioritization of bioaccumulable pharmaceutical substances discharged in hospital effluents. J. Environ. Manag..

[3] Gogoia A., Mazumderb P., Tyagi V.K., Chaminda G.G.T., An A.K., Kumar M. (2018). Occurrence and fate of emerging contaminants in water environment: A review. Groundw. Sustain. Dev..

[4] Ebele A.J., Abdallah M.A.-E., Harrad S. (2017). Pharmaceuticals and personal care products (PPCPs) in the freshwater aquatic environment. Emerg. Contam..

[5] Gadipelly C., Pérez-González A., Yadav G.D., Ortiz I., Ibáñez R., Rathod V.K., Marathe K.V. (2014). Pharmaceutical industry wastewater: Review of the technologies for water treatment and reuse. Ind. Eng. Chem. Res..

[6] Saucier C., Karthickeyan P., Ranjithkumar V., Lima E.C., dos Reis G.S., de Brum I.A.S. (2017). Efficient removal of amoxicillin and paracetamol from aqueous solutions using magnetic activated carbon. Environ. Sci. Pollut. Res..

[7] Styszko K., Nosek K., Motak M., Bester K. (2015). Preliminary selection of clay minerals for the removal of pharmaceuticals, bisphenol A and triclosan in acidic and neutral aqueous solutions. C. R. Chim..

[8] Suna K., Shia Y., Wang X., Li Z. (2017). Sorption and retention of diclofenac on zeolite in the presence of cationic surfactant. J. Hazard. Mater..

[9] Song J.Y., Bhadra B.N., Jhung S.H. (2017). Contribution of H-bond in adsorptive removal of pharmaceutical and personal care products from water using oxidized activated carbon. Microporous Mesoporous Mater..

[10] Muthanna J.A. (2017). Adsorption of non-steroidal anti-inflammatory drugs from aqueous solution using activated carbons: Review. J. Environ. Manag..

[11] Ahmed M.B., Zhou J.L., Ngo H.H., Guo W., Chen M. (2016). Progress in the preparation and application of modified biochar for improved contaminant removal from water and wastewater. Bioresour. Technol..

[12] Fernandez M.E., Ledesma B., Roman S., Bonelli P.R., Cukierman A.L. (2015). Development and characterization of activated hydrochars from orange peels as potential adsorbents for emerging organic contaminants. Bioresour. Technol..

[13] Ncibi M.C., Sillanpää M. (2017). Optimizing the removal of pharmaceutical drugs Carbamazepine and Dorzolamide from aqueous solutions using mesoporous activated carbons and multi-walled carbon nanotubes. J. Mol. Liq..

[14] Bhadra B.N., Ahmed I., Kim S., Jhung S.H. (2017). Adsorptive removal of ibuprofen and diclofenac from water using metalorganic framework-derived porous carbon. Chem. Eng. J..

[15] Bandosz T.J., Ania C.O., Bandosz T.J. (2006). Surface Chemistry of Activated Carbons and Its Characterization, Activated Carbons Surfaces in Environmental Remediation.

[16] Zhou C.H., Zhang D., Tong D.-S., Wu L.-M., Yu W.-H., Ismadii S. (2012). Paper-like composites of cellulose acetate organo-montmorillonite for removal of hazardous anionic dye in water. Chem. Eng. J..

[17] Lee S.Y., Kim S.J. (2002). Adsorption of naphthalene by HDTMA modified kaolinite and halloysite. Appl. Clay Sci..

[18] Deng L., Yuan P., Liu D., Annabi-Bergaya F., Zhou J., Chen F., Liu Z. (2017). Effects of microstructure of clay minerals, montmorillonite, kaolinite and halloysite, on their benzene adsorption behaviors. Appl. Clay Sci..

[19] Anadao P., Pajolli I.L.R., Hildebrando E.A., Wiebeck H. (2014). Preparation and characterization of carbon/montmorillonite composites and nanocomposites from waste bleaching sodium montmorillonite clay. Adv. Powder Technol..

[20] Fuertes A.B., Sevilla M. (2015). High-surface area carbons from renewable sources with a bimodal micro-mesoporosity for high-performance ionic liquid-based supercapacitors. Carbon.

[21] Myronyuk I.F., Mandzyuk V.I., Sachko V.M., Gun’ko V.M. (2016). Structural Features of Carbons Produced Using Glucose, Lactose, and Saccharose. Nanoscale Res. Lett..

[22] Bakandritsos A., Kouvelos E., Steriotis T., Petridis D. (2005). Aqueous and Gaseous Adsorption from Montmorillonite-Carbon Composites and from Derived Carbons. Langmuir.

[23] Chen L.-F., Liang H.-W., Lu Y., Cui C.-H., Yu S.-H. (2011). Synthesis of an Attapulgite Clay@Carbon Nanocomposite Adsorbent by a Hydrothermal Carbonization Process and Their Application in the Removal of Toxic Metal Ions from Water. Langmuir.

[24] Wu X., Zhu W., Zhang X., Chen T., Frost R.L. (2011). Catalytic deposition of nanocarbon onto palygorskite and its adsorption of phenol. Appl. Clay Sci..

[25] Wu X., Gao P., Zhang X., Jin G., Xu Y., Wu Y. (2014). Synthesis of clay/carbon adsorbent through hydrothermal carbonization of cellulose on palygorskite. Appl. Clay Sci..

[26] Wu X., Xu Y., Zhang X., Wu Y., Gao P. (2015). Adsorption of low-concentration methylene blue onto a palygorskite/carbon composite. New Carbon Mater..

[27] Joussein E., Petit S., Churchman G.J., Theng B.K.G., Righi D., Delvaux B. (2005). Halloysite clay minerals-a review. Clay Miner..

[28] Yuan P., Tan D., Annabi-Bergaya F. (2015). Properties and applications of halloysite nanotubes: Recent research advances and future prospects. Appl. Clay Sci..

[29] Wu X., Liu C., Qi H., Zhang X., Dai J., Zhang Q., Zhang L., Wu Y., Peng X. (2016). Synthesis and adsorption properties of halloysite/carbon nanocomposites and halloysite-derived carbon nanotubes. Appl. Clay Sci..

[30] Jiang L., Zhang C., Wei J., Tjiu W., Pan J., Chen Y., Liu T. (2014). Surface Modifications of Halloysite Nanotubes with Superparamagnetic Fe_3_O_4_ Nanoparticles and Carbonaceous Layers for Efficient Adsorption of Dyes in Water Treatment. Chem. Res. Chin. Univ..

[31] Jin J., Fu L., Yang H., Ouyang J. (2015). Carbon hybridized halloysite nanotubes for high-performance hydrogen storage capacities. Sci. Rep..

[32] Zhang J., Liu T., Liu M. (2018). Hydrothermal synthesis of halloysite nanotubes@carbon nanocomposites with good biocompatibility. Microporous Mesoporous Mater..

[33] Zhang Y., Ouyang J., Yang H. (2014). Metal oxide nanoparticles deposited onto carbon-coated halloysite nanotubes. Appl. Clay Sci..

[34] http//chem.nlm.nih.gov/chemidplus/.

[35] Szczepanik B., Słomkiewicz P., Garnuszek M., Czech K., Banaś D., Kubala-Kukuś A., Stabrawa I. (2015). The effect of chemical modification on the physico-chemical characteristics of halloysite: FTIR, XRF, and XRD studies. J. Mol. Struct..

[36] Brunauer S., Emmett P.H., Teller E. (1938). Adsorption of gases in multimolecular layers. J. Am. Chem. Soc..

[37] Gregg S.J., Sing K.S.W. (1982). Adsorption. Surface Area and Porosity.

[38] Lim C.K., Bay H.H., Noeh C.H., Aris A., Majid Z.A., Ibrahim Z. (2013). Application of zeolite-activated carbon macrocomposite for the adsorption of Acid Orange 7: Isotherm. kinetic and thermodynamic studies. Environ. Sci. Pollut. Res..

[39] Jedynak K., Szczepanik B., Rędzia N., Słomkiewicz P., Kołbus A., Rogala P. (2019). Ordered Mesoporous Carbons for Adsorption of Paracetamol and Non-Steroidal Anti-Inflammatory Drugs: Ibuprofen and Naproxen from Aqueous Solutions. Water.

[40] Kodama S., Sekiguchi H. (2006). Estimation of point of zero charge for activated carbon treated with atmospheric pressure non-thermal oxygen plasmas. Thin Solid Films.

[41] Langmuir I. (1916). The constitution and fundamental properties of solids and liquids. J. Am. Chem. Soc..

[42] Freundlich H.M.F. (1906). Über die adsorption in lősungen. Z. Phys. Chem..

[43] Temkin M.I., Pyzhev V. (1940). Kinetics of ammonia synthesis on promoted iron catalysts. Acta Physicochim. USSR.

[44] Sips R. (1948). Combined form of Langmuir and Freundlich equations. J. Chem. Phys..

[45] Petrushenko I.K., Petrushenko K.B. (2019). Physical adsorption of hydrogen molecules on single-walled carbon nanotubes and carbon-boron-nitrogen heteronanotubes: A comparative DFT study. Vacuum.

[46] De Souza T.N.V., de Carvalho S.M.L., Gurgel M., Vieira A., da Silva M.G.C., Brasil D.D.S.B. (2018). Adsorption of basic dyes onto activated carbon: Experimental and theoretical investigation of chemical reactivity of basic dyes using DFT-based descriptors. Appl. Surf. Sci..

[47] Thonhauser T., Cooper V.R., Li S., Puzder A., Hyldgaard P., Langreth D.C. (2007). Van der Waals density functional: Self-consistent potential and the nature of the van der Waals bond. Phys. Rev. B.

[48] Tamijani A.A., Salam A., de Lara-Castells M.P. (2016). Adsorption of Noble-Gas Atoms on the TiO_2_(110) Surface: An Ab Initio-Assisted Study with van der Waals-Corrected DFT. J. Phys. Chem. C.

[49] Grimme S. (2006). Semiempirical GGA-type density functional constructed with a long-range dispersion correction. J. Comput. Chem..

[50] Vydrov O.A., Voorhis T.V. (2010). Nonlocal van der Waals density functional: The simpler the better. J. Chem. Phys..

[51] Berland K., Cooper V.R., Lee K., Schröder E., Thonhauser T., Hyldgaard P., Lundqvist B.I. (2015). van der Waals forces in density functional theory: A review of the vdW-DF method. Rep. Prog. Phys..

[52] Pearson R.G. (1986). Absolute electronegativity and hardness correlated with molecular orbital theory. Proc. Natl. Acad. Sci. USA.

[53] Yildizhan G., Caliskan S., Ozturk R. (2018). Palladium and platinum based solid and hollow nanoparticles: An ab-initio study of structural and electronic properties. J. Solid State Chem..

[54] Li Z.Q., Lu C.J., Xia Z.P., Zhou Y., Luo Z. (2007). X-ray diffraction patterns of graphite and turbostratic carbon. Carbon.

[55] Joussein E., Petit S., Delvaux B. (2007). Behaviour of halloysite clay under formamide treatment. Appl. Clay Sci..

[56] Cheng H., Frost R.L., Yang J., Liu Q., He J. (2010). Infrared and infrared emission spectroscopic study of typical Chinese kaolinite and halloysite. Spectrochim. Acta A Mol. Biomol. Spectrosc..

[57] Sing K.S.W., Everett D.H., Haul R.A.W., Moscou L., Pierotti R.A., Rouquerol J., Siemieniewska T. (1985). Reporting physisorption data for gas/solid systems with special reference to the determination of surface area and porosity. Pure Appl. Chem..

[58] Bernal V., Erto A., Giraldo L., Moreno-Piraján J.C. (2017). Effect of solution pH on the adsorption of paracetamol on chemically modified activated carbons. Molecules.

[59] Lagregren S. (1898). About the theory of so-called adsorption of soluble substances. Kungl. Sven. Veten. Akad. Handl..

[60] Ho Y.S., McKay G. (1999). Pseudo-second-order model for sorption processes. Process Biochem..

[61] Weber W.J., Morris J.C. (1963). Kinetics of adsorption on carbon solution. J. Sanit. Eng. Div. Am. Soc. Civ. Eng..

[62] Komers R., Tomanova D., Beranek L. (1973). Adsorption of Weak Bases from the Gas Phase on Organic Ion-Exchangers. J. Catal..

[63] Słomkiewicz P.M. (2004). Determination of the adsorption model of alkenes and alcohols on sulfonic copolymer by inverse gas chromatography. J. Chromatogr. A.

